# Mitochondrial dysfunction in hearing loss: Oxidative stress, autophagy and NLRP3 inflammasome

**DOI:** 10.3389/fcell.2023.1119773

**Published:** 2023-02-20

**Authors:** Peipei Li, Shen Li, Le Wang, Hongmin Li, Yang Wang, Hongbing Liu, Xin Wang, Xiaodan Zhu, Zhangsuo Liu, Fanglei Ye, Yuan Zhang

**Affiliations:** ^1^ Department of Integrated Traditional and Western Nephrology, The First Affiliated Hospital of Zhengzhou University, Zhengzhou, China; ^2^ Henan Province Research Center for Kidney Disease, Zhengzhou, China; ^3^ Department of Neurology, The First Affiliated Hospital of Zhengzhou University, Zhengzhou, China; ^4^ Department of Otology, The First Affiliated Hospital of Zhengzhou University, Zhengzhou, China

**Keywords:** oxidative stress, autophagy, NLRP3 inflammasome, hearing loss, mitochondrial dysfunction

## Abstract

Sensorineural deafness becomes an inevitable worldwide healthy problem, yet the current curative therapy is limited. Emerging evidences demonstrate mitochondrial dysfunction plays a vital role of in the pathogenesis of deafness. Reactive oxygen species (ROS)-induced mitochondrial dysfunction combined with NLRP3 inflammasome activation is involved in cochlear damage. Autophagy not only clears up undesired proteins and damaged mitochondria (mitophagy), but also eliminate excessive ROS. Appropriate enhancement of autophagy can reduce oxidative stress, inhibit cell apoptosis, and protect auditory cells. In addition, we further discuss the interplays linking ROS generation, NLRP3 inflammasome activation, and autophagy underlying the pathogenesis of deafness, including ototoxic drugs-, noise- and aging-related hearing loss.

## 1 Introduction

Hearing loss affects almost 5% of the world’s population and impacts people ranges of all ages (Sheffield and Smith, 2019). Hearing loss causes communication barriers between people, leading to isolation, depression, dementia, and other psychological problems. This primary effect not only impairs the quality life of patients themselves but also causes indirect economic losses to society due to the reduced productivity caused by communication difficulties (Cunningham and Tucci, 2017). Therefore, a comprehensive understanding mechanism of hearing loss is extremely important for disease prevention and treatment.

**FIGURE 1:**
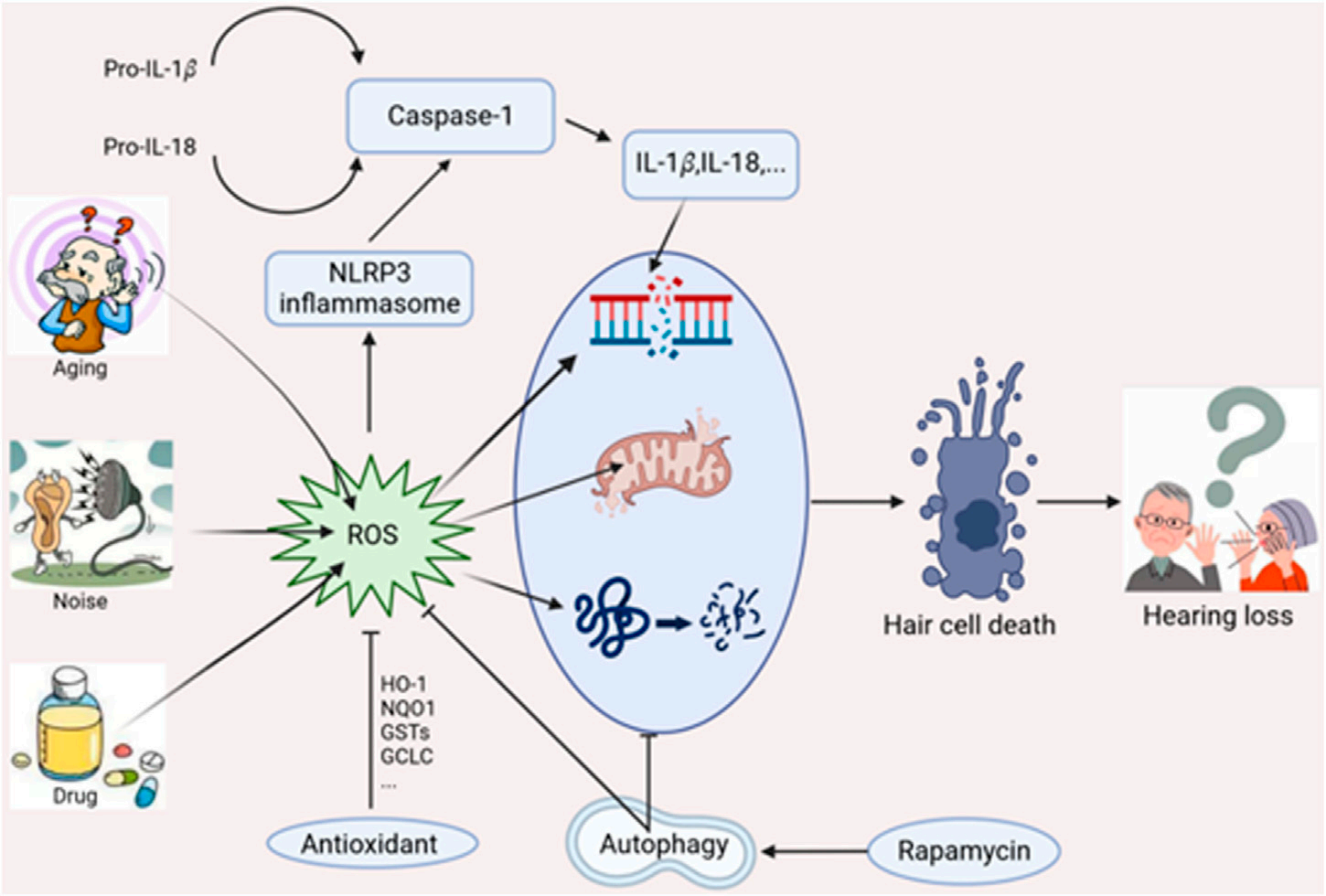
Interplay of oxidative stress, autophagy and NLRP3 inflammasome in hearing loss.

Hearing loss is mainly considered a sensory disorder in humans. Multiple factors contribute to the pathogenesis of sensorineural hearing loss (SNHL), such as noise exposure, ototoxic drugs (aminoglycoside antibiotics, platin-based anticancer drugs, and loop diuretics), genetic mutations, aging, and chronic conditions. Histopathological changes of SNHL are characterized by mechanosensory hair cell damage, spiral ganglion neuron (SGN) loss, and stria vascularis atrophy (Keithley, 2020; Rousset et al., 2020). Emerging studies have suggested that mitochondrial DNA damage, reactive oxygen species (ROS) overproduction, and inflammatory mediators activation are associated with subsequent cochlear damage. Mitochondria ROS could induce inflammasome activation that promotes various disease progression (Martinon, 2010; Sorbara and Girardin, 2011). Moreover, ROS could also induce cellular defense process such as autophagy, a cytoprotective manner that deliver damaged organelles to lysosomes for degrader (Wang and Klionsky, 2003; Vernon and Tang, 2013). Current studies reveal autophagy exhibits an antioxidative capacity to protect against hair cell damage and possesses the potential to alleviate noise-induced hearing loss (NIHL) (Ye et al., 2019). This review mainly discusses the underlying mechanism affecting cochlear damage and hearing loss, including ROS-induced oxidative stress, autophagy, and NLRP3 inflammasome (Figure 1).

## 2 Mitochondria and reactive oxygen species

### 2.1 ROS generation

ROS, such as superoxide anions, hydrogen peroxide, and hydroxyl radicals, are reactive molecules containing oxygen and are mainly generated in the mitochondria (Han and Someya, 2013; Shrestha et al., 2021). Various stress conditions could markedly induce mitochondrial ROS production, increase metabolic rates, hypoxia, and membrane damage. The mitochondrial electron transport chains generate the electrochemical gradient to drive oxidative phosphorylation for ATP synthesis. The electron leakage in the respiratory chain results in the production of superoxide (Chance et al., 1979; Turrens, 2003; Brand, 2010). Seven precise sites have been identified for mitochondrial ROS production, complex I (NADH), complex III (cytochrome c oxidoreductase), glycerol 3-phosphate dehydrogenase, NADH-Q oxidoreductase, pyruvate dehydrogenase, and 2-oxoglutarate dehydrogenase. Of these sites, complex I-III are well shown to display the maximum capacities to produce ROS (Turrens et al., 1985; Muller et al., 2004; Pryde and Hirst, 2011). Antioxidant enzymes (catalase, SOD, and glutathione peroxidase) exert the cytoprotective effect by scavenging ROS. Oxidative stress represents an imbalance between ROS production and the antioxidant defense system. Overproduction of ROS can destroy biological membranes, attack DNA, cause gene mutations and protein denaturation, and ultimately cause various human diseases, neurodegenerative diseases, carcinogenesis, and aging-related diseases (Fujimoto and Yamasoba, 2019).

### 2.2 Mitochondrial ROS in hearing loss

Mitochondria ROS overproduction plays a key role in cochlear damage and hearing impairment (Pryde and Hirst, 2011). ROS-induced oxidative stress cause direct damage in cochlear hair cells, SGNs, and stria vascularis. ROS generation also leads to inflammation and pro-inflammatory cytokines secretion (Wong and Ryan, 2015). There are approximately 16,000 hair cells, and 35,000 transmissive SGNs in the human cochlea (Ehret and Frankenreiter, 1977; Schettino and Lauer, 2013). The cochlear hair cell and SGN refer to the foundation of sensory, and they do not regenerate after death. The most common histological sign of SNHL is cochlear sensory cell loss and damage (Schuknecht et al., 1973).

Oxidative stress induced by ROS can cause NIHL (Choi and Choi, 2015). ROS production is immediately detected in the cochlear tissue after exposure to high-intensity sound, and could be detected for several days (Ohlemiller et al., 1999). ROS-induced lipid peroxidation (malondialdehyde and 4-hydroxynonenal) can lead to apoptosis and vasoactive lipid peroxidation, and reduce cochlear blood flow (Yamashita et al., 2004; Fetoni et al., 2019). Noise-related ischemia and reperfusion further potentiate ROS generation (Wong and Ryan, 2015). In addition, noise trauma could induce elevate mitochondrial calcium levels and metabolic demand in hair cells, subsequently increasing ROS production (Henderson et al., 2006; Peng and Jou, 2010). Moreover, the antioxidants have been shown to attenuate NIHL when given either before or after noise exposure (Le et al., 2017). Ototoxic drugs are general associated with ototoxicity in clinical application. Hair cells are mechanoreceptors uniquely containing mechanotransducer (MET) channels on stereo ciliary bundles (Beurg et al., 2009; Wagner and Shin, 2019). Aminoglycoside transports into the hair cell *via* the MET channel, results in cell demise (Guthrie, 2008). Cisplatin-induced hearing loss is mainly caused by inflammation. Inflammation leads to the overproduction of NADPH oxidases subunits, impaired antioxidant defense systems. The subsequent ROS accumulation results in cell death in multiple manners, apoptosis, autophagy, pyroptosis, and necroptosis (Sheth et al., 2017; Gentilin et al., 2019; Nan et al., 2019). Cisplatin induced chronic changes affect outer hair cells, stria vascularis, and SGNs (Meech et al., 1998; Alam et al., 2000; Bowers et al., 2002). The free radical theory of aging believes that ROS attacking life macromolecules and causing tissue and cell damage is the fundamental cause of body aging (Beckman and Ames, 1998). Aging stress causes mitochondrial DNA damage, ROS overproduction, antioxidant function decreasing, and subsequent cochlear senescence (Fujimoto and Yamasoba, 2014). Mouse models of age-related hearing loss (ARHL) are known to harbor excessive ROS levels mitochondrial DNA mutations and. Increased ROS levels leads to lower mitochondrial membrane potential, affecting hair cell survival and hearing loss (Han and Someya, 2013; Yamasoba et al., 2013). SOD1-null mice display premature ARHL due to hair cell loss (McFadden et al., 1999).

In summary, noise trauma, ototoxic drugs or aging could first induce elevate mitochondrial calcium levels and metabolic demand, or lead to ischemia and reperfusion, or causes mitochondrial DNA damage. Then ROS is overproduced and leads to lower mitochondrial membrane potential, lipid peroxidation, pro-inflammatory cytokines secretion. Finally, senescence and cell death occur in the cochlea.

### 2.3 Antioxidants strategy in hearing loss

Antioxidants have the function of scavenging ROS and can be used to treat oxidative stress-related hearing loss. Mitochondrial-targeted antioxidants are expected to prevent or treat mitochondria-related disorder (Fujimoto and Yamasoba, 2019). Currently, the novel effective antioxidants, MitoQ, and SkQR1 exhibit protective effects against hearing loss in mouse auditory cell lines and animal models. MitoQ is a ubiquinone derivative that covalently binds to lipophilic triphenylphosphine (TPP) ions through aliphatic carbon chains and targets mitochondria (Kelso et al., 2001). The efficacy of mitochondria target antioxidants is more relying on their ability to cross the phospholipid bilayer and mitochondrial ROS elimination. The positive charge and hydrophilicity of TPP cation enable MitoQ accumulate to several hundred-fold in negatively charged mitochondria (Murphy and Smith, 2007). SkQ1 and SkQR1 are designed from MitoQ and display greater permeability in membrane transportation than MitoQ (Antonenko et al., 2008). The two antioxidants could also inhibit mitochondrial ROS formation. Treatment with MitoQ or SkQR1 protects against gentamicin-induced ototoxicity in animal models (Jankauskas et al., 2012; Ojano-Dirain et al., 2014). However, the efficacy of MitoQ in human studies is limited (Snow et al., 2010), and more clinical trials are required to evaluate its therapeutic effects on hearing loss patients. In addition, another antioxidant astaxanthin exerts powerful activities for ROS scavenging due to its unique membrane function and ability to permeate the blood-brain barrier (Nan et al., 2019). Collectively, these novel antioxidants may be a feasible method to alleviate and prevent ROS-related hearing loss.

## 3 Antioxidative role of mitochondria autophagy on hearing loss

### 3.1 Mitochondria autophagy mechanism

Autophagy is the process through which cells degraded cytoplasmic contents in the lysosome. Despite autophagy is once considered a non-selective process that mediated the bulk degradation of cytoplasmic components, current studies have demonstrated it can specifically target damaged organelles, such as mitochondria, ruptured lysosomes, peroxisomes, ER, lipid droplets (Levine and Kroemer, 2019). More than 40 autophagy-related (ATG) genes have been reported in yeast, of which *ATG11* and *ATG101* are considered core genes. These autophagic factors are recruited at the initiation, elongation, and closure of autophagosome. Mostly, the cellular cargo either containing an LC3-interacting region (LIR) or labeled with a ubiquitin tag could be recruited to adaptor proteins, which serves as a bridge between substances and LC3 (or GABARAP) motif and conjugate to the autophagosome membrane. Moreover, specific proteins bind to tripartite motif (TRIM) members for alternative autophagic degradation (Kimura et al., 2017). During the past decade, emerging studies have reported the involvement of autophagy in human diseases, particularly in neurodegenerative disorders, autoimmune diseases, and cancers (Mizushima and Levine, 2020).

The selective elimination of abnormal mitochondria *via* the autophagy pathway is termed mitophagy. Mitochondria degradation is triggered under the conditions of basal mitochondrial quality control, dysfunction, and developmental processes (Twig et al., 2008; Sato and Sato, 2011). The most well-studied mitophagy pathways are termed ubiquitin- and receptor-mediated mitophagy. The first pathway is mainly involved in PINK/Parkin, while receptor mitophagy includes NIX/BNIP3L, BNIP3, and FUNDC1. PINK1, a serine/threonine-protein kinase, is mainly associated with oxidative stress, autophagy, and apoptosis in cell (Trempe et al., 2013). Physiological PINK1 is transported into mitochondria *via* the mitochondrial target signal (MTS) and the membrane potential (Δ*Ψ* m) (Yamano and Youle, 2013). The whole length of PINK1 is cleaved by matrix processing peptidase and PINK-associated rhomboid-like protease (PARL), releasing into the cytoplasm and degraded through ubiquitin-proteasome system (Deas et al., 2011). PINK1 transportation to the inner mitochondrial membrane is impaired in damaged or depolarized mitochondrial, causing the PINK1 accumulation on the outer mitochondrial to form dimers. Phosphorylation of PINK1 dimers recruits Parkin (an E3 ubiquitin ligase) through direct interaction, further activating Parkin and initiating mitophagy (Sarraf et al., 2013; Gladkova et al., 2018). Damaged mitochondria are enclosed into phagosome, and delivered to lysosome for depredating, as well as the PINK1 and Parkin protein degradation (Li et al., 2022a).

Bcl-2 and adenovirus E1B 19-kDa-interacting protein 3 (BNIP3) and BNIP3-like (BNIP3L/NIX) are homologous members of the Bcl-2 family, which are initially reported as pro-apoptotic proteins (Imazu et al., 1999). Both of the proteins are expressed on the mitochondria outer membrane, and contain classical LIR domains as essential components for mitophagy initiation. NIX/BNIP3L is required for excess mitochondria removal during reticulocyte maturation (Schweers et al., 2007). Phosphorylation of BNIP3 and NIX at serine residue sites near the LIR motif could stabilize their interactions with LC3, and promote mitophagy (Zhu et al., 2013; Rogov et al., 2017).

FUNDC1 is another well-known mitophagy receptor localized to the outer membrane of mitochondria. Similar to BNIP3 and BNIP3L, FUNDC1 also contains an LIR motif for interacting with the LC3 region (Liu et al., 2012). Under normal condition, FUNDC1 is phosphorylated at Try18 and Ser 13 by Src kinase and CK2, respectively. Hypoxia stress causes FUNDC1 dephosphorylation *via* inhibiting Src kinase and CK2 activity (Liu et al., 2012). Dephosphorylated FUNDC1 has a significantly higher affinity to LC3. Moreover, phosphoglycerate mutase family member 5 (PGAM5) also response for FUNDC1 dephosphorylation under hypoxia or mitochondrial uncoupling (Chen et al., 2014).

### 3.2 Mitochondria ROS and autophagy

The complex interplay between mitochondria oxidative stress and autophagy has been extensively reported. Excessive ROS level triggers general autophagy over mitophagy (Frank et al., 2012), while moderate ROS level triggers mitophagy through specific signaling activation. In turn, the redox signaling with mitophagy possess a cytoprotective protective effect to promote cell survival (Zhang et al., 2021). Dynein-related protein 1 (DRP-1) is the key factor in controlling mitochondria division and mitophagy initiation. Inhibiting DRP-1-dependent mitophagy can cause damaged mitochondria accumulation and ATP metabolic dysfunction, leading to cochlear hair cell senescence (Lin et al., 2019).

Increased ROS levels can regulate mitophagy *via* several pathways, NF-κB, mTOR, p38-MAPK, SIRT, etc., For example, ROS (H2O2) accumulation results in NF-κB inhibitor releasing in H2O2 oxidation manner, which activate NF-κB signaling (Sies et al., 2017). NF-κB promotes mitophagy through upregulating p62 expression, and attenuates NLRP3 inflammasome-mediated mitochondrial damage. p38-MAPK belong to the MAPK family and is induced by stress stimuli, such as inflammatory cytokines and oxidative stress. p38-MAPK affect the Parkin-mediated mitophagy in Parkin/PINK1-dependent pathway (Xiao et al., 2017). Sirtuins are a family of NAD-dependent deacetylases, which is response to activated NAD + function. SIRT1 affects Parkin translocation to mitochondrial inner membrane, and is slightly related to NAD +/NADH ratio alterations (Di Sante et al., 2015). SIRT1 could deacetylate FOXO1/3 and enhances mitophagy *via* activating the PINK1-Parkin axis (Zhao et al., 2021a). Inhibition of miR-34a/SIRT1 signaling enhance mitophagy and attenuate ROS-related hair cell death in the hearing loss context, implicating complicated interplay between ROS and mitophagy (Xiong et al., 2019).

### 3.3 Autophagy impairment contributes to ototoxic drugs, noise exposure, and aging-related hearing loss

Autophagy is an essential process participating in normal cochlear development and normal function of inner ear cells. Several autophagic genes (e.g., ATG4, ATG5) are expressed in the mouse cochlea from the embryonic phage until the adult phage (Aburto et al., 2012). *ATG4*-null mice show the common pathological features of the inner ear (Mariño et al., 2010). Deletion of Atg5 results in HCs degeneration and severe congenital hearing loss in mice due to the accumulation of undesired autophagic substrates (Fujimoto et al., 2017).

Dysfunction autophagy is linked to ototoxic hearing loss. Autophagic protein formation in the early stage of cisplatin treatment exerts cytoprotective activity, and induce HEI-OC1 cell death in the late stage of cisplatin treatment (Youn et al., 2015; Li et al., 2018). Neomycin treatment could induce HC death *via* mitophagy suppression (He et al., 2017; Zhang et al., 2022). Systemically aminoglycoside administration preferentially induces cochlear hair cell death and results in irreversible hearing loss. Gentamicin triggers RIPOR2 translocation from cochlear stereocilia to the pericuticular area in murine hair cells; RIPOR2 interacts with the autophagic protein GABARAP to activate autophagy, resulting in hair cells death (Li et al., 2022c). Downregulated RIPOR2 or GABARAP could prevent hair cell death and alleviate hearing loss in mice, suggesting autophagy components may be therapeutic targets to prevent ototoxicity (Li et al., 2022b).

Presbycusis is a common sensory disorder associated with aging. Autophagy deficiency occurs both in the aging model of HEI-OC1 cells and cochlear explant cultures (He et al., 2020). Furthermore, SGN from premature deaf mice model also display autophagic decreasing and accumulated lipofuscin (Menardo et al., 2012). Upregulation of autophagy promotes aging HC survival and slows the degeneration of auditory cells (Xiong et al., 2019). Rapamycin, an mTOR inhibitor, enhances SGNs autophagy *via* inhibiting the mTOR pathway, resulting in ARHL amelioration (Liu et al., 2022). Moreover, mitophagy refers to a specific autophagy process for damaged mitochondria degradation and cellular homeostasis maintaining. Mitophagy proteins (PINK1/Parkin, and BNIP3) is downregulated in the mouse auditory cortex and inferior colliculus region with aging (Youn et al., 2020); additionally, colocalization of autophagosome and lysosome is also decreased in the auditory system of aged mouse, indicating mitophagy impairment in the central auditory system.

ROS-induced oxidative damage is a major element of NIHL. The interplay between autophagy and ROS generation in NIHL has been detected. TTS-noise induced low-level oxidative stress activates autophagy that exerts a protective effect on outer hair cell survival, while excessive oxidative stress overwhelms the beneficial potential of autophagy, leading to outer hair cell death (Yuan et al., 2015). This point is supported by the results: noise-induced oxidative marker elevations is noise-dose-dependent in outer hair cells; whereas, autophagy marker is sharply increased after TTS, but slightly elevated in PTS and unaltered in sPTS noise (Yuan et al., 2015). Moreover, several antioxidant proteins or autophagic activators display the capacity to alleviate NIHL. SESN2 (sestrin 2) is an endogenous antioxidant protein. SESN2 interact with Unc-51-like protein kinase 1 (ULK1) to promote Beclin1 phosphorylation, Parkin mitochondrial translocation, and further facilitate mitophagy (Kumar and Shaha, 2018), indicating it might be as a therapeutic target against noise-induced cochlear injury. Pejvakin is a peroxisome-associated protein from the gasdermin family and exhibit a protective effect against harmful oxidative stress. Pejvakin-mediated selective autophagic degradation (pexophagy) could protect auditory hair cells against noise-induced damage *via* modulating the recruitment of autophagosome-associated protein MAP1LC3B (LC3B) (Defourny et al., 2019). Calcineurin inhibitor FK506 is also reported as an autophagic activator. It can activate autophagy *via* binding to ATPase catalytic subunit in neuronal cells, and alleviate neurodegenerative diseases (Kim et al., 2017). Treatment with FK506 (tacrolimus) could also reduce noise-induced hair cell damage *via* activating autophagy, and alleviated NIHL in adult CBA/J mice (He et al., 2021).

## 4 NLRP3 inflammasome in hearing loss

Inflammasomes are likely responsible for elevated ROS production in immune cells. The cochlea was once thought to have immune privileges. However, the immune privileged status changed when it was discovered that lymphocytes, such as macrophages can infiltrate into the endolymphatic sac of guinea pigs (Watson et al., 2017).

## 4.1 NLRP3 inflammasome activation

NLRP3 inflammasome is the most studied inflammasome comprised of NLRP3, an apoptosis associated speck-like protein containing a CARD (ASC) and procaspase (Agostini et al., 2004). NLRP3 contains an N-terminal pyrin domain (PYD), nucleotide-binding oligomerization (NACHT) domain, and C-terminal leucine-rich repeat (LRR) domain. Under the healthy condition, NLRP3 displays auto-repressed *via* the internal interaction of the NACHT and LRRs domain. PAMPs from microorganisms or DAMPs from endogenous lead to the removal of auto-repressed (Bryant and Fitzgerald, 2009). Exposure of the PYD domain mediates ASC and pro-caspase one recruitment, triggering the caspase-1 activation, and pro-inflammatory cytokines maturation (such as IL-1β and IL-18). NLRP3 is a general sensor of cellular stress that could be activated by ROS proximity to the inflammasome. NLRP3 inflammasome activation has been tightly regulated, and participates in several physiological processes, including immune system response and host defenses. The activated mechanisms included three panels, ionic flux, lysosomal damage, and ROS-mediated mitochondrial dysfunction (Tschopp and Schroder, 2010).

### 4.2 NLRP3 inflammasome in hearing loss

Mitochondria ROS has been confirmed as the crucial mechanism of ototoxic drugs. However, it is undesirable for the clinical application of antioxidants to alleviate the cisplatin-induced hearing loss. Cisplatin can trigger the assembly of NLRP3 inflammasome, induce marginal cell pyroptosis and cochlear damage (Yu et al., 2022). The pathological morphology changes and NLRP3 expression could be suppressed by inhibiting the upstream signal TXNIP. In addition, the NLRP3 inflammasome activation triggered by ROS was also reported in the ARHL and NIHL. The mechanisms are mainly involved in downstream inflammatory cytokines secretion (Shi et al., 2017; Zhuang et al., 2018; Sai et al., 2022).

Gain-of-function mutation in *NLRP3* causes a spectrum of autoinflammatory diseases termed cryopyrin-associated periodic syndromes (CAPS) (Yu and Leslie, 2011). Abnormal NLRP3 inflammasome activation and excessive IL-1β secretion is the major reason of CAPS, and has been demonstrated in three clinical subtypes: neonatal-onset multisystem inflammatory disease (NOMID), Muckle-Wells syndrome (MWS), and familial cold autoinflammatory syndrome (FCAS). These phenotypes share general features, recurrent fever, rash, headache, etc., (Moltrasio et al., 2022). Hearing loss is one of the most common symptoms of NOMID and MWS, but is rare in FCAS. Notably, postcontrast MRI examination observed pathologic cochlear enhancement in most NOMID and MWS patients (Ahmadi et al., 2011), indicating the blood-labyrinth barrier is permeable by inflammation. Genetic studies of CAPSs have reported more than 80 *NLRP3* variants, the majority of which are missense mutations located in exon 3, encoding the conserved NACHT domain (Conforti Andreoni et al., 2011). Whereas, minority NLRP3 mutations in other regions (e.g., LRR domain) are related to syndromic and non-syndromic hearing loss, such as deafness autosomal dominant 34 (DFN34) and keratitis fugax hereditaria (KFH) diseases. DFNA34 caused by the missense substitution p.Arg918Gln (c.2753G > A) in exon 7 (encoding the LRR domain) has been reported in two unrelated families (Nakanishi et al., 2017). Subjects in one family exhibited hearing loss accompanied by autoinflammatory features, while another family patients displayed hearing loss segregated without any other organ symptoms. Pathologic cochlea enhancement is identified in these subjects, implicating the cochlear inflammation in hearing loss. The hearing loss in several members is alleviated or completely resolved after treatment by IL-1β inhibitor anakinra (Nakanishi et al., 2017). A further study demonstrated the NLRP3 inflammasome were also activated in inner ear macrophages, and cochlea-infiltrated macrophages contribute to NLRP3-related hearing loss in the murine model (Nakanishi et al., 2017; Nakanishi et al., 2020).

### 4.3 Interplay between autophagy, NLRP3 inflammasome, and ROS generation

ROS induces NLRP3 inflammasome and results in cell damage. The mitophagy/autophagy system may eliminate mitochondrial ROS, thereby inhibiting the of NLRP3 inflammasome activation (Zhou et al., 2011). Autophagy can protect cells from inflammatory damage by inhibiting the activation of the inflammasome and pro-inflammatory signaling pathways (He et al., 2017). However, under starvation conditions of yeast, autophagy can promote inflammation *via* ATG-5 dependent non-classical manner (Dupont et al., 2011; Cao et al., 2019). In turn, NLRP3 inflammasome exerts an inhibitory effect on autophagy by cleaving the downstream signal molecule TRIF (Lai et al., 2018). Moreover, NLRP3 inflammasome activation mediate IL-1β releasing, which represents the main neurotoxicity in neuronal diseases, including brain stroke, Parkinson, and Alzheimer’s Disease (Wang et al., 2017; Han et al., 2019; Zhao et al., 2021b).

## 5 Another mechanisms of hearing loss

ERG (ether-a-go-go-related gene) channels are the members of the voltage-dependent potassium channel family, which have three subtypes, as ERG1 (Kv 11.1), ERG2 (Kv 11.2), and ERG3 (Kv11.3). The results of Ramazan Bal et al. show that the ERG channels appear to contribute to setting action potential (AP) frequency, threshold for AP induction, and, possibly, resting membrane potentials in this cells, which plays an important role in the formation of hearing (Yildirim and Bal, 2018).

Acid-sensing ion channels (ASICs) are voltage-independent and proton-gated channels. Bao-Ming Wu and Tian-Dong Leng demonstrate that oxidative stress increases ASIC1a expression/activation through the JNK signaling pathway, which may provide insight into the pathogenesis of neurological disorders that involve both ROS and activation of ASIC1a (Cakir et al., 2019; Wu et al., 2021).

Oxidative stress-induced Ca2+ permeable transient receptor potential melastatin 2 (TRPM2) channels are expressed at high levels in the brain, which seems to link neuronal excitability to cellular metabolism and participate in the pathogenesis of neurodegenerative disorders, such as SNHL (Bal et al., 2020). Activation of TRPM2 by reactive oxygen/nitrogen species (ROS/RNS) occurs following the production of ADPR, an intracellular activator. TRPM2 activation has been associated with cell death in the presence of increased oxidative and nitrosative stress, such as during oxygen–glucose deprivation. In the cochlear nucleus (CN), among these redox-sensitive TRP channels, TRPM2 is a potential candidate sensor of oxidative stress.

All in all, these studies have shown that oxidative stress-induced ion channels, including TRPM2 cation channels (Bal et al., 2020), ASICs (Cakir et al., 2019), ATP-sensitive potassium channels (Bal et al., 2018) and ERG channels (Yildirim and Bal, 2018), play an important role in hearing loss. These channels seem to transduce the increase in levels of reactive oxygen species exceeding physiological limits into specific cellular responses, such as triggering the apoptosis pathway, and ultimately leading to hearing loss.

## 6 Conclusion

In summary, ROS-mediated mitochondrial dysfunction combined with NLRP3 inflammasome activation contribute to progression of neurodegenerative diseases, including SNHL. ROS-induced oxidative stress and NLRP3-activated pro-inflammatory cytokines can damage the cochlea structure of auditory hair cells and SGNs. Novel efficient antioxidants can remove ROS and protect auditory cells. In addition, autophagy not only eliminate damaged proteins and organelle but also reduce ROS formation, and alleviate hearing loss. In most conditions, autophagy can ameliorate inflammatory diseases *via* inhibiting the NLRP3 inflammasome activation. Whereas, the pro-inflammatory effect of autophagy should be also noted in some cases. There remain some details to be solved. For example, the potential regulatory mechanism of autophagy in NLRP3 inflammasome remain unclear in SNHL progression. Moreover, intervention time in inflammatory reactions in the early or advanced stages of hearing loss should also be investigated. These conclusions provide therapeutic targets for inner ear diseases, such as targeting mitochondrial ROS, neutralizing pro-inflammatory cytokines, and appropriately increasing autophagy levels.

## Abbreviations

ATG, autophagy-related gene; ARHL, age-related hearing loss; DAMP, damage-associated molecular pattern; FCAS, familial cold autoinflammatory syndrome; LIR, LC3-interacting region; MET, mechanotransducer; MRI, magnetic resonance diffusion tension imaging; MWS, Muckle-Wells syndrome; NAD, nicotinamide adenine dinucleotide; NIHL, noise-induced hearing loss; LC3B, microtubule-associated protein one light chain 3β; PAMP, Pathogen associated molecular pattern; ROS, reactive oxygen species; SNHL, sensorineural hearing loss; SODs, superoxide dismutase; SGNs, spiral ganglion neurons; NOMID, neonatal-onset multisystem inflammatory disease; ULK1, unc-51-like protein kinase 1; 3MA, 3-methyladenine.
